# Intrinsic functional connectivity reduces after first-time exposure to short-term gravitational alterations induced by parabolic flight

**DOI:** 10.1038/s41598-017-03170-5

**Published:** 2017-06-12

**Authors:** Angelique Van Ombergen, Floris L. Wuyts, Ben Jeurissen, Jan Sijbers, Floris Vanhevel, Steven Jillings, Paul M. Parizel, Stefan Sunaert, Paul H. Van de Heyning, Vincent Dousset, Steven Laureys, Athena Demertzi

**Affiliations:** 1 Antwerp University Research Centre for Equilibrium and Aerospace (AUREA), University of Antwerp, Antwerp, Belgium; 2 Vision Lab, Department of Physics, University of Antwerp, Antwerp, Belgium; 3 Department of Radiology, Antwerp University Hospital & University of Antwerp, Antwerp, Belgium; 4 KU Leuven – University of Leuven, Department of Imaging & Pathology, Translational MRI, Leuven, Belgium; 5 University of Bordeaux, CHU de Bordeaux, INSERM Magendie, Bordeaux, France; 6 Coma Science Group, GIGA-Research & Neurology Department, University and University Hospital of Liège, Liège, Belgium; 7 Institut du Cerveau et de la Moelle Epinière - Brain and Spine Insititute, Hôpital Pitié-Salpêtrière, Paris, France

## Abstract

Spaceflight severely impacts the human body. However, little is known about how gravity and gravitational alterations affect the human brain. Here, we aimed at measuring the effects of acute exposure to gravity transitions. We exposed 28 naïve participants to repetitive alterations between normal, hyper- and microgravity induced by a parabolic flight (PF) and measured functional MRI connectivity changes. Scans were acquired before and after the PF. To mitigate motion sickness, PF participants received scopolamine prior to PF. To account for the scopolamine effects, 12 non-PF controls were scanned prior to and after scopolamine injection. Changes in functional connectivity were explored with the Intrinsic Connectivity Contrast (ICC). Seed-based analysis on the regions exhibiting localized changes was subsequently performed to understand the networks associated with the identified nodes. We found that the PF group was characterized by lower ICC scores in the right temporo-parietal junction (rTPJ), an area involved in multisensory integration and spatial tasks. The encompassed network revealed PF-related decreases in within- and inter-hemispheric anticorrelations between the rTPJ and the supramarginal gyri, indicating both altered vestibular and self-related functions. Our findings shed light on how the brain copes with gravity transitions, on gravity internalization and are relevant for the understanding of bodily self-consciousness.

## Introduction

Spaceflight induces several physiological changes in the human body, such as fluid shifts, neurovestibular disturbances, bone loss and muscle atrophy^1^. Space crew adapt fairly well to these changes, depending on the site of action and the applied countermeasures. Yet, despite several decades of human spaceflight, countermeasures are not entirely successful. For example, space motion sickness is still present among several space travellers when arriving in the International Space Station, and upon return to Earth, orthostatic intolerance often occurs next to spatial disorientation, continued osteoporosis and muscle atrophy^1, 2^. Some space travellers adapt easier to the relatively hostile environment of space than others, and second - time fliers certainly experience fewer problems, which has been well described for e.g. space motion sickness^2^.

The central nervous system also seems capable of adaptation to microgravity by the process of neuroplasticity, as previously shown in animals^3–5^. Yet, little is known about the effects of microgravity and gravity transitions on the human brain^6^. Recently, in a functional MRI study with a single cosmonaut, we showed that long-duration spaceflight induced functional changes in the right insula and in sensorimotor-cerebellar connectivity^7^.

However, research on humans in space is expensive and subject to several logistic and payload restrictions. Hence, ground-based models have been developed, in which some aspects of spaceflight can be simulated. Dry immersion^8^ and head-down bed rest^9^ mimic certain characteristics of spaceflight quite well, such as ‘supportlessness’ or fluid shifts respectively. Nevertheless, these space analogs are performed in the presence of a constant gravitational force, and therefore not suitable to study the impact of microgravity and acute gravitational transitions on the human brain.

**Figure 1:**
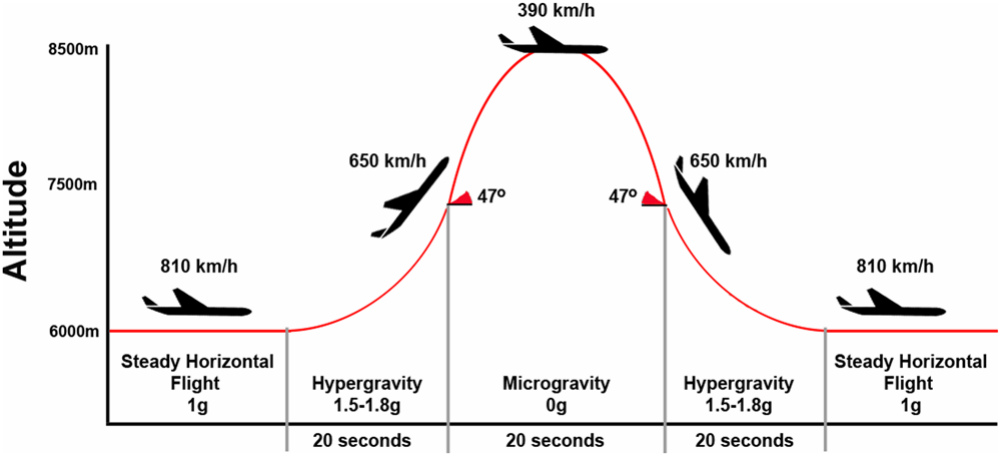
Flight trajectory during the parabolic manoeuvre.

Parabolic flight is a “ground-based” alternative, during which a specific parabolic trajectory is carried out, wherein the acceleration of the aircraft cancels the gravity acceleration. A hypergravity phase, characterized by 1.5–1.8 g lasting around 30–35 s, precedes and follows the microgravity phase. Microgravity resembles zero g and lasts around 20–25 s. In between parabolas, the aircraft flies in normal 1 g conditions^10^. The sensation of weightlessness is caused when the aircraft is in free fall, because during that time it does not exert any ground reaction force on its contents. Thus, PF consists of gravity transitions, (microgravity, hypergravity and normal gravity phases), generated during 31 parabolas. An entire flight lasts around 3 to 3.5 h (Fig. 1).

The aim of the present study was to measure the effects of acute exposure to gravitational transitions, induced by PF, in naïve human subjects. We hypothesized that a) after short-term exposure to gravity alterations the brain will show modifications at the functional level and b) these modifications will concern the vestibular system. Our first hypothesis is supported by previous fMRI studies showing changes in brain function after acute environmental modulations (e.g. refs 11–13) or after intense subjective experiences (e.g. refs 14 and 15), suggesting that short-term plasticity is measurable with neuroimaging tools. Our second hypothesis is based on the observations that, in normal conditions, vestibular cues give information on the pull of the gravito-inertial acceleration being the vector sum of gravity and all other linear accelerations exerted on the vestibulum. Together with somatosensory information from joints, skin and muscles, the vestibular information is integrated with allocentric information from the visual surroundings and egocentric cues about one’s own body axis^16^. During a PF, especially during the microgravity phase, the vestibular input is largely disturbed and therefore might cause an incongruity with the normal terrestrial expectations regarding verticality and spatial orientation^17^. As no previous neuroimaging investigations have been performed under these conditions^6, 18, 19^, a data-driven approach was here implemented to investigate changes in fMRI functional connectivity during resting state. Functional connectivity refers to the temporal correlation between spatially remote neurophysiological events, expressed as a deviation from statistical independence in distributed brain regions^20^. In order to better comprehend how the identified regions, which exhibit these changes, are related to the rest of the brain, we subsequently estimated the cerebral networks correlating with these nodes. In order to debrief overall functionality, subjective ratings on the level of wakefulness, emotional function and motion sickness were collected prior and after the PF.

## Materials and Methods

### Subjects

The inclusion criteria to participate in the PF were: adult participants, no previous participation in a PF, non-smokers, and good physical condition according to a complete medical check-up screening. Each selected subject participated in one PF only. Based on these criteria, an initial cohort of 31 volunteers was obtained. Three subjects were excluded from the analysis because post-flight fMRI data could not be obtained due to logistical reasons. The final PF cohort included 28 healthy participants (11 female; mean (SD) age 31 (7) years). Prior to the PF, all selected participants received scopolamine (0.25 mg/1 mL; 0.7 mL for males and 0.5 mL for females), a muscarinic receptor antagonist known to alleviate motion sickness^21^. To account for the effects of the drug, an independent control group of 12 adults (4 female; mean (SD) age 24 (3) years) who received scopolamine was also included. These participants had no previous experience with PFs.

All participants provided a signed informed consent form. The study was approved by the local ethics committee of the Antwerp University Hospital (13/38/357), by the European Space Agency (ESA) medical board and by the Comité de Protection des Personnes Nord Ouest III (Caen, France). All clinical investigations have been conducted according to the principles expressed in the Declaration of Helsinki.

### Procedure

The PFs took place during the European Space Agency (ESA) PF campaigns on board of the Airbus A-300 Zero G, in April 2014 (60^th^ campaign) and September 2014 (61^st^ campaign); and on board of the Airbus A-310 Zero G in May 2015 (1^st^ cooperative CNES/DLR/ESA PF campaign) and June 2015 (62^th^ campaign). All flights departed from Bordeaux-Merignac airport (France) and were exploited by Novespace (www.novespace.fr). Each campaign consisted of PFs on 3 consecutive days. Each PF included 31 parabolic manoeuvers at zero g. Every parabola started with a pull-up phase and ended with a pull-out phase at 1.8 g, both lasting about 20 s. The duration of the zero-g condition was about 21 s (Fig. 1). Every flight lasted approximately three hours in total.

Approximately one hour before take-off, all participants were administered a subcutaneous injection of scopolamine by the campaign medical doctor as is routinely the case in PFs^22^. On board, subjects were seated and secured with a safety belt during the first 5 parabolas, to enhance adaptation to the peculiar sensation of gravity shifts. Afterwards, they were allowed to free-float in a therefore restricted zone for at least 5 consecutive parabolas. A pre-flight scanning session took place 1 to 2 days before the flight and the post-flight session was performed right after (<4 hours) the completion of the flight, at the University of Bordeaux and University Hospital of Bordeaux (France).

Prior to and immediately after PF, participants fulfilled standardized questionnaires assessing the level of wakefulness, emotional function and motion sickness^23^. The Epworth Sleepiness Scale^24^ (ESS) is a 8-item scale ranging from 0–3, which assesses sleepiness and it was incorporated to assess the possible fatigue and drowsiness associated with the administration of scopolamine; a score of 10 separates between normal individuals and excessive daytime sleepiness. The Positive and Negative Affect Scale (PANAS)^25^ is a 20-item measure of positive and negative affect ranging from 1 to 5; momentary mean scores for the Positive Affect Score is 29.7 (7.9) and for the Negative Affect Score is 14.8 (5.4). The Motion Sickness Assessment Questionnaire (MSAQ)^26^ is a 16-item questionnaire comprising of four subscales, all assessing a different aspect of motion sickness (gastrointestinal, central, peripheral and sopite-related). The Misery Scale (MISC)^27^ is an 11-point scale ranging from 0 to 10 which measures the level of motion sickness; each participant had to report a MISC score 7 times in total: pre-flight (seated in the plane before take-off), after the 1^st^, 6^th^, 10^th^, 20^th^ and 30^th^ parabola and post-flight (right after landing). Questionnaire data were also collected in the scopolamine control group in the same way. Data were analysed with SPSS v21 (IBM Corp, Armonk, New York). Bonferroni-corrected Wilcoxon Signed Rank tests were performed to test differences in scoring between pre- and post PF, as well as between pre- and post- scopolamine intake in the control group.

### Data acquisition

PF group: pre- and post-flight data were acquired on a 3 T GE MR 750 W (GE Healthcare, Milwaukee, Wisconsin, USA) MRI scanner at the University of Bordeaux and University Hospital of Bordeaux (France), using a 32-channel head coil. During resting state, 280 multislice T2*-weighted images were acquired with a gradient-echo echo-planar imaging sequence using axial slice orientation and covering the whole brain (voxel size = 3 × 3 × 3 mm; matrix size = 64 × 64 × 42; repetition time = 2 s; echo time = 20 ms; flip angle = 77°; field of view = 192 × 192 mm). For anatomical reference, a high-resolution T1-weighted image was acquired for each subject (T1-weighted 3D magnetization-prepared rapid gradient echo sequence).

Scopolamine control group (non-PF): two scanning sessions took place, a baseline medication-free session and 3 hours after the administration of scopolamine (Antwerp University Hospital, Belgium). Pre- and post-scopolamine data were acquired on a 3 T Siemens MAGNETOM Prisma scanner (Siemens, Erlangen, Germany), using a 32-channel head coil. During the resting state scanning period, an identical MRI sequence was used as for the PF group.

### Data analysis

Data preprocessing was performed with Statistical Parametric Mapping 12 (SPM12; www.fil.ion.ucl.ac.uk/spm) and statistical analysis with the CONN v.16 functional connectivity toolbox (www.nitrc.org/projects/conn). The initial three volumes were discarded to avoid T1 saturation effects. Preprocessing steps included slice-time correction, realignment, segmentation of structural data, normalization into standard stereotactic Montreal Neurological Institute (MNI) space and spatial smoothing using a Gaussian kernel of 6 mm full width at half-maximum (FWHM). Motion correction further encompassed motion artifact detection and rejection using the artifact detection toolbox (ART; http://www.nitrc.org/projects/artifact_detect). Specifically, an image was defined as an outlier image if the head displacement in x, y, or z direction was greater than 0.5 mm from the previous frame, or if the rotational displacement was greater than 0.02 radians from the previous frame, or if the global mean intensity in the image was greater than 3 SDs from the mean image intensity for the entire resting session. Outliers in the global mean signal intensity and motion were subsequently included as nuisance regressors within the first-level general linear model. For noise reduction, we used the anatomical component-based noise correction method aCompCor^28^. This approach models the influence of noise as a voxel-specific linear combination of multiple empirically estimated noise sources by deriving principal components from noise regions of interest (ROIs) and by including them as nuisance parameters within first-level general linear model. Specifically, the anatomical image for each participant was segmented into white matter (WM), gray matter, and cerebrospinal fluid (CSF) masks. To minimize partial voluming with gray matter, the WM and CSF masks were eroded by one voxel, which resulted in substantially smaller masks than the original segmentations^29^. The eroded WM and CSF masks were then used as noise ROIs. Signals from the WM and CSF noise ROIs were extracted from the unsmoothed functional volumes to avoid additional risk of contaminating WM and CSF signals with gray matter signals. A temporal band-pass filter of 0.008–0.09 Hz was applied. Residual head motion parameters (three rotation and three translation parameters, plus another six parameters representing their first-order temporal derivatives) were also regressed out.

Statistical analysis adopted a hypothesis-free (voxel-to-voxel) approach. First-level voxel-to-voxel analysis encompassed the estimation of voxel-to-voxel functional correlation matrix within each subject. From the residual BOLD time series at every voxel within an a priori GM mask (isotropic 2-mm voxels) the matrix of voxel-to-voxel bivariate correlation coefficients was computed^30^. From this voxel-to-voxel correlation matrix, the intrinsic connectivity contrast (ICC) was computed^31^. The ICC characterizes the strength of the global connectivity pattern between each voxel and the rest of the brain. In short, the ICC is based on network theory’s degree metric, which represents the number of voxels showing a correlation with each other voxel. Therefore, a whole-brain map is produced wherein the intensity of each voxel reflects the degree to which that voxel is connected to the rest of the brain. In order to avoid the need of an arbitrary correlation threshold, an ICC power map is finally created representing the average r^2^ connectivity of a given voxel and all the other above threshold voxels, with a greater ICC score representing greater average strength of the correlations in a given voxel. This method of hypothesis-free exploration of connectivity changes has been previously employed by others^7, 31–35^. Second-level group analysis utilized a 2 × 2 repeated measures design, with “Group” (PF, non-PF) as between-subject factor, further modelling the effect of scanning site, and “Condition” (Pre-flight scan, Post-flight scan) as within-subject factor. To disentangle the effects observed in the PF group as opposed to the effects attributed to scopolamine, a conjunction was carried out between the post-flight decreases in connectivity in the PF as compared to the non-PF group, and the pre-flight compared to post-flight decreases in connectivity in the PF group only, i.e.: [PF > non-PF Pre < Post negative contrast] & [PF Pre < Post negative contrast]. Due to the unbalanced design, two supplementary analyses were performed in order to increase statistical power and ensure validity and interpretation of the results: connectivity analyses with PF participants matched for age and gender to the non-PF group and bootstrapping (Supplementary Material).

As the ICC is an explorative metric which localizes changes in functional connectivity related to the experimental modulations, classic region of interest-based fMRI connectivity analysis on the regions exhibiting changes was performed to understand the networks associated with the identified nodes^31^. For these seed regions, time-series from the contained voxels were averaged together. This averaged time-series was used to estimate whole-brain correlation r maps, which were then converted to normally distributed Fisher’s z transformed correlation maps to allow for subsequent group-level analysis. Non-parametric permutation tests assessed the distributions of each experimental group or condition.

## Results

### Questionnaires

Out of the 28 included participants, 24 fulfilled the PANAS, ESS, MSAQ and MISC questionnaires. Data from 4 participants was not assessed due to logistical reasons. Based on the self-reports, participants did not experience severe motion sickness, abnormal positive/negative affect or sleepiness (Table 1). After the PF, there was a decrease in negative affect (PANAS subscale) compared to preflight (Z = −3.99, p < 0.001). For the PANAS positive affect and the ESS, there were no differences. After scopolamine intake, there was a decrease in positive affect (PANAS subscale) compared to pre-scopolamine (Z = −2.606, p = 0.009). No differences were found for the PANAS negative affect subscale and the ESS questionnaire.

**Table 1:** Subjective ratings on the Positive and Negative Affect Scale, the Epworth Sleepiness questionnaire, Motion Sickness Assessment Questionnaire and Misery Scale scores (mean (SD)).

	Pre flight (n = 24)	During flight* (n = 24)	Post flight (n = 24)	Pre scop (n = 12)	During scop (n = 12)	Post-scop (n = 12)
PANAS, Positive Affect subscale	35 (4)		35 (6)	32 (6)		29 (7)
PANAS, Negative Affect subscale	16 (4)		12 (5)	12 (2)		11 (1)
Epworth Sleepiness Scale	7 (3)		8 (5)	6 (3)		9 (4)
MSAQ, total (in %)			23 (17)			22 (8)
MSAQ, central subscale (in %)			20 (16)			27 (14)
MSAQ, sopite-related subscale (in %)			22 (20)			31 (15)
MSAQ, gastro-intestinal subscale (in %)			29 (27)			17 (7)
MSAQ, peripheral subscale (in %)			20 (18)			15 (6)
Misery Scale score	0.5 (0.8)	1.2 (1.3)	0.5 (0.8)	0.0 (0.0)	1.0 (0.2)	0.5 (0.7)

MSAQ: Motion Sickness Assessment Questionnaire; PANAS: Positive and Negative Affect Scale; PF: parabolic flight; scop: scopolamine; SD: standard deviation.

*Averaged over the 5 assessments during the flight.

### Connectivity analysis

**Figure 2:**
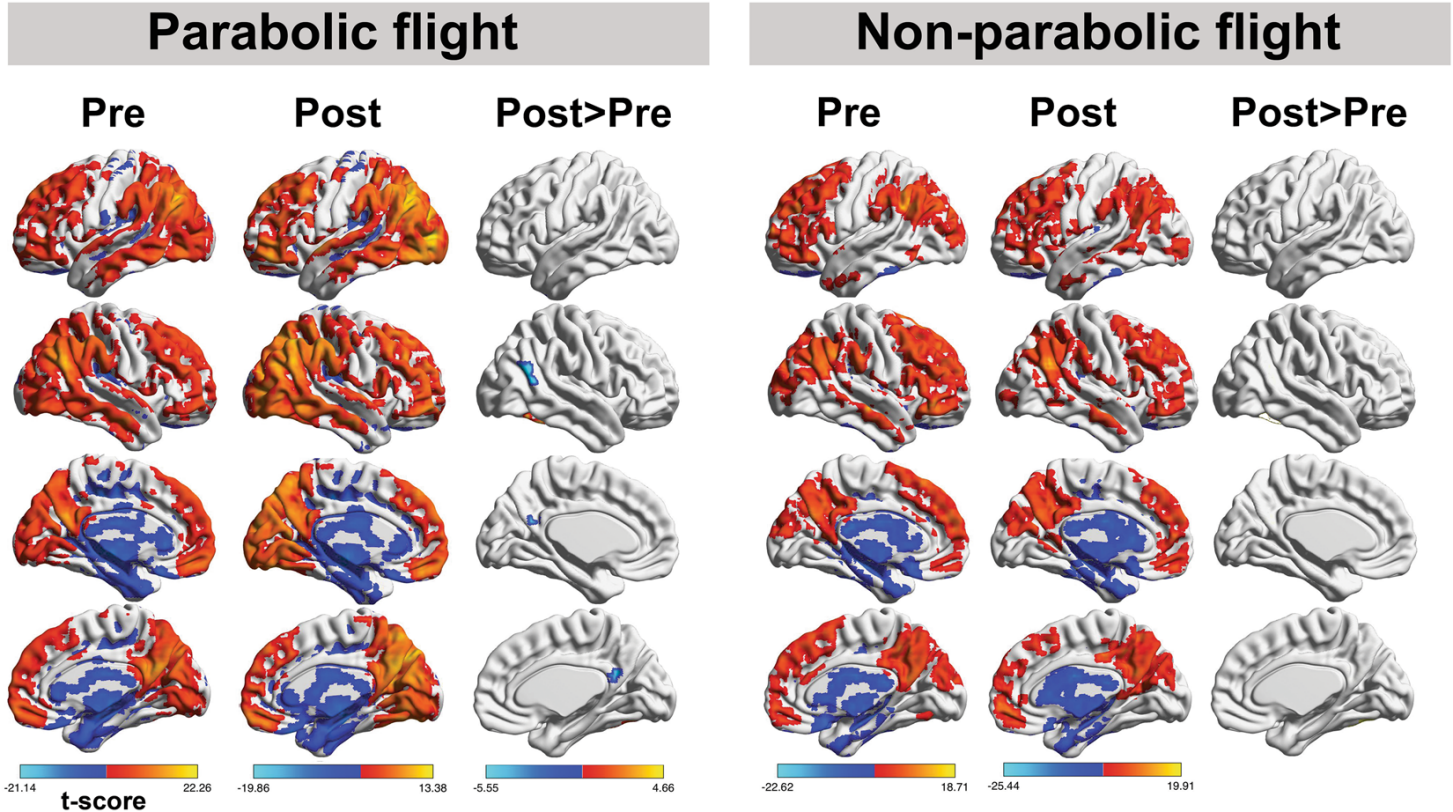
Group-level explorative analysis was performed to localize modifications in global connectivity before and after participation to the parabolic flight (n = 28) and before and after scopolamine intake in the non-parabolic flight group (n = 12). The spatial patterns represent average maps of higher (red) and lower (blue) intrinsic connectivity contrast scores, with a greater score representing greater average strength of the correlations in a given voxel. Statistical maps are rendered on a surface template and are thresholded at cluster-level family wise error rate p < 0.05 (two-sided, permutation testing).

**Figure 3:**
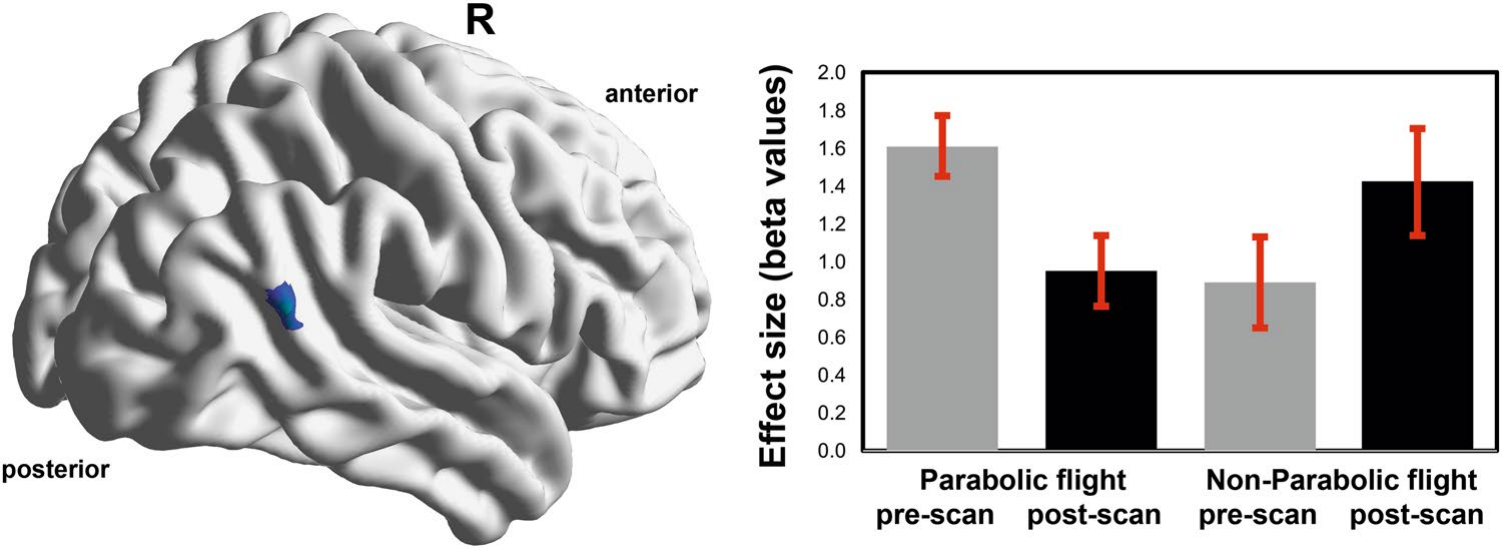
The hypothesis-free exploration of connectivity changes points to the right angular gyrus/temporo-parietal junction as the region with lower scores on the intrinsic connectivity contract, implying that this region has decreased participation in whole-brain connectivity at post-flight scan. The map represents the results of the conjunction analysis, suggesting that the effect can be attributed to the parabolic flight group as compared to the non-parabolic flight control group. Bars indicate effect sizes (beta values) and error bars 90% CI in the same cluster.

In terms of motion, four outlier images were detected in the PF group and none in the non-PF group. For the ICC explorative analysis, the main effects of each group at pre- and post-scan are summarized in Fig. 2. For the PF group, between-condition differences were identified in posterior cingulate cortex and right parietal gyrus. For the non-PF group, no post-pre scan differences in ICC connectivity were found (Fig. 3). The interaction analysis revealed that the modification of the connectivity pattern was observed in the right temporo-parietal junction (rTPJ)/angular gyrus (rAG) in the PF group in comparison to the non-PF group, at post-scan as compared to pre-scan assessment (T(38) = −3.32, p < 0.001 FWE cluster-level, permutation testing; cluster size: 260 voxels, peak coordinate x, y, z = [58–64 18]). The rAG was also identified after the conjunction analysis (cluster size: 148 voxels, x, y, z = [57–66 25]; Fig. 3), and the two sub-analyses with the age and gender-matched groups and bootstrapping (Supplementary Material).

**Figure 4:**
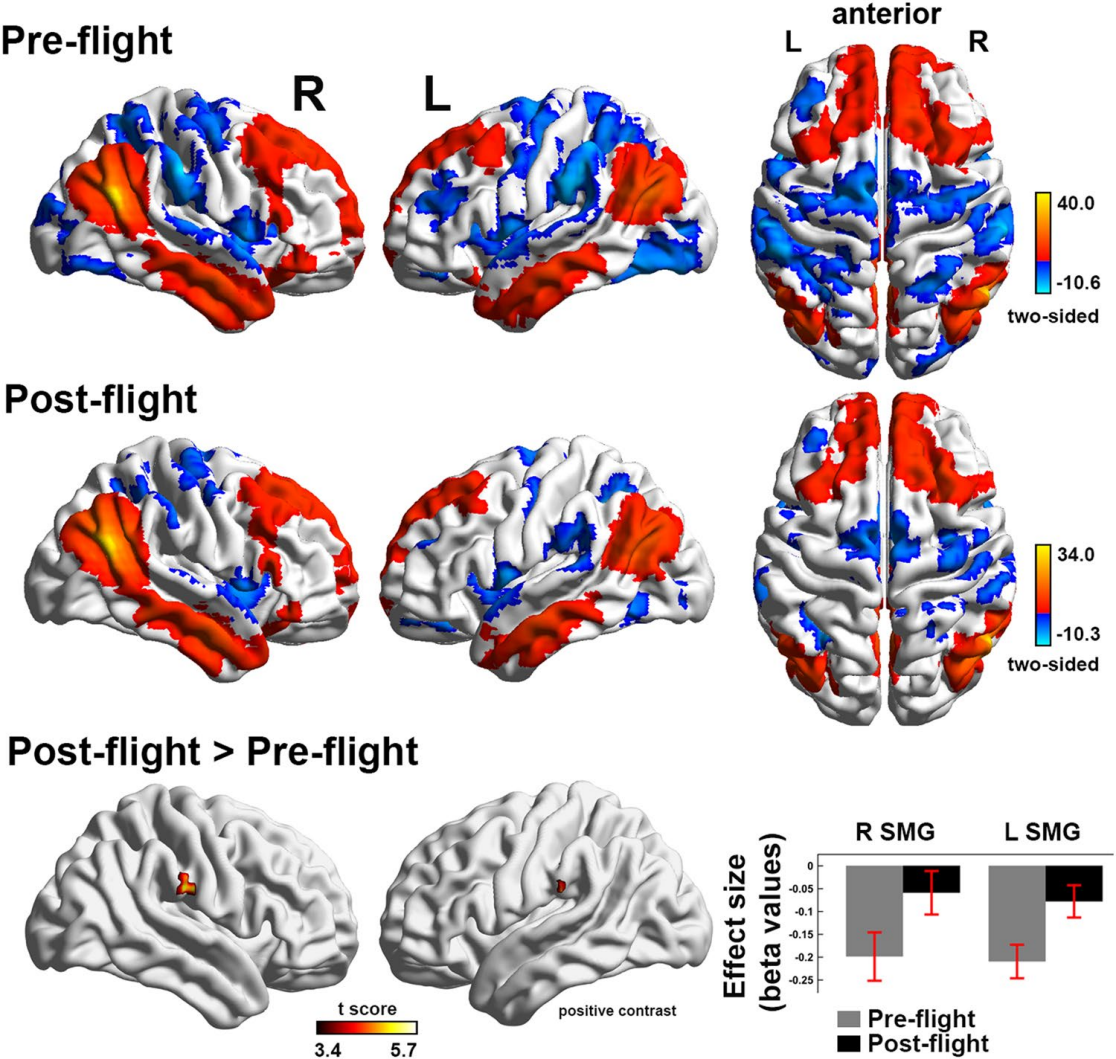
The networks encompassed by the right angular gyrus/temporo-parietal junction in the parabolic flight group. The default mode network (DMN; red) and the DMN anticorrelated regions (blue) were the set of areas that were functionally connected with the right angular gyrus at pre- and post-flight. The between-condition differences in the encompassed networks included fewer anticorrelations with bilateral supramarginal gyri. The statistical maps are rendered on a surface template and have been thresholded at cluster-level family wise error rate p < 0.05 (permutation tests). Bars indicate cluster-level effect sizes (beta values) and error bars 90% CI.

The rAG identified by the interaction analysis was then used as a seed area to perform classic region of interest analysis in order to better comprehend the network associated with the identified node. Both in pre-flight and post-flight scan the encompassed areas showing positive connectivity were located in lateral parietal, superior/middle frontal gyri, superior/middle/inferior temporal gyri as well as mesio-frontal, posterior parietal/precuneal regions and cerebellum. Negative connectivity was identified in bilateral supramarginal gyri/SMA, superior frontal gyri and temporal/temporo-occipital/lateral occipital regions (Fig. 4). Between-condition contrast pointed to fewer negative correlations (anticorrelations) with R supramarginal (SMG; BA 40; 63–24 31) and L supramarginal gyri (SMG; BA 40; −59–31 36) (FWE p < 0.05 cluster-level, permutation testing) at post- compared to pre-flight scan.

## Discussion

The aim of the present study was to assess functional connectivity changes after short-term acute exposure to gravitational alterations. Hereto, we exposed healthy naïve individuals to a PF and measured resting-state fMRI connectivity before and after the exposure to altered gravitational forces. With no a priori assumptions, we found a decrease of the ICC scores in the rTPJ/rAG after the PF. These results suggest the rAG/TPG has reduced participation in whole-brain connectivity after short-term exposure to altered gravity, most possibly related to changes in vestibular function. For instance, in order to maintain gaze stabilization, postural control and spatial orientation, the human brain integrates visual, somatosensory and vestibular input signals^36^. In the vestibular system, angular accelerations are detected by the semicircular canals, while linear accelerations, and thus gravity, are sensed by the otolith organs (i.e. utriculus and sacculus). Therefore, due to the alterations in gravitational force, the afferent information from the otolith organs is significantly altered. This gives rise to a loss of congruence between visual, proprioceptive and vestibular input and the loss of the otherwise tight coupling of canal-otolith information in the presence of Earth’s gravity. As a result, the vestibular system and its functions are challenged thoroughly.

Previous investigations also suggest that the rAG is involved in the processing and integration of vestibular, visual and proprioceptive input^37^. For example, inhibition of the right TPJ caused difficulties with the perception of the upright and maintaining an internal representation of verticality^38, 39^. Also, disruptive TMS to the AG showed that this region mediated the interaction between visuo-proprioceptive weighting and realignment^40^. Past studies further point to the involvement of the TPJ in timing of interception of an object^41^. With regards to the effects of gravity, it was found that the attempt to intercept an object accelerated by gravity had a reverse response pattern in weightlessness as compared to normal gravity^42, 43^. In the same line, an fMRI study, assessing the perception of moving objects according to natural or reversed gravity, found the engagement of the TPJ and insula, suggesting an internalization of gravity in these regions^44^. Indeed, when a sensory mismatch occurs, e.g. between proprioceptive and visual information, the brain can rearrange the two modalities, by calibrating the modality which receives the lowest weight^45^. The correlation between the weight of a modality and the extent of calibration during a mismatch was found to be disrupted when TMS temporarily inactivated the AG^40^. Taken together, these studies suggest an important role of the TPJ in the integration of multisensory modalities for achieving optimal vestibular function. During a PF, there is a constant shift in gravity levels provoking many conflicting sensory signals, such as proprioception^10, 46^. The latter occurs in combination with visual disturbed impressions when floating upside down during the microgravity phase. This can hamper verticality and perception of self-location^47–50^.

Important as the rAG may be for vestibular processing, it is commonly accepted that the human brain does not possess a unique primary vestibular cortex and that vestibular information is processed in a distributed network (e.g. refs 51 and 52). From electrophysiological and tracer studies in primates, as well as neuroimaging studies in humans, we know that the so-called “vestibular cortex” encompasses the TPJ and posterior insula, the somatosensory cortex, the posterior parietal cortex, the anterior insula, and the lateral and medial frontal cortices^53^. Specifically, the parieto-insular vestibular cortex (PIVC) in primates is considered as the “hot spot” of vestibular processing^51, 52^ which presumably maps to the parietal operculum (OP2) in humans^54^. However, the exact location of the human analogue of the PIVC remains controversial^51^. The fact that this key region was not identified in our analysis can only be speculated. Future hypothesis-driven explorations of the vestibular cortex may shed more light on the preferential contribution of each region to the PF experience.

Interestingly, the TPJ has been considered important for bodily self-consciousness^50, 55^, i.e., the non-conceptual and pre-reflective processing and representation of body-related information^56^. With regards to self-location (“where I am in space”), previous studies with patients with epileptic seizures and focal electrical stimulation of the TPJ elicited sensations of body tilt and altered gravity. Interestingly, the AG and TPJ have been reported to be involved in out-of-body experiences, which can be considered as a deficient perception of self-location and self-being, when this area is inactivated or lesioned^49, 57–61^. Furthermore, reports from people experiencing microgravity (either during a PF or during spaceflight) have elucidated that the absence of gravity can elicit several illusory own-body perceptions, e.g. the inversion illusion (i.e. the feeling of the body being upside-down relative to the extrapersonal space or vice versa)^62^. Such experience is considered to be the result of a combination of altered gravitational input, a multisensory disintegration and top-down modulation^55^. Considering the above, it is possible that the here identified reduced rAG connectivity implies the inability to compute and correct the conflicting sensory inputs it receives during microgravity.

Our results also resonate with lesion studies, pointing to the right hemisphere for elaborating an internal model of verticality and controlling body orientation^63^. For example, patients with Pusher syndrome (a disorder of postural balance that manifests as a pushing away toward the contralesional side in unilateral stroke) were shown to depend predominantly on otolith inputs when they sustained right hemispheric lesions^47^. This indicates an asymmetrical otolith mechanism, concurrent with a dominance of the right non-dominant hemisphere in processing vestibular cues^64^. Also, a PET study in patients with a unilateral vestibular neuritis showed that the vestibular graviceptive deficits in these patients (as measured by the deviation on the subjective visual vertical) correlate positively with regional glucose metabolism in the right hemisphere^65^. This highlights not only the lateralized dominance of the cortical vestibular network, but also the functional dominance in verticality perception and the processing of gravitational cues, resonating with our findings.

We also found decreases in the anticorrelated connectivity between the rAG/rTPJ and bilateral SMG after the flight. Anatomically, the AG and SMG are connected through arcuate (u-shaped) connections within the same hemisphere^66^. The SMG plays a well-established role in vestibular function, as shown by studies implementing both caloric^44, 64, 67–70^ and galvanic vestibular stimulation^71, 72^. Additionally, the SMG plays a distinct role in the perception of verticality^38^. Apart from this within-hemispheric connection, there is both a structural and functional interhemispheric connectivity between the AG and SMG^73^. Interestingly, the rAG is part of the default mode network (DMN) while the SMG are part of a set of areas classically anticorrelating to the DMN^74, 75^. Such anticorrelated connectivity has been associated with cognitive function^76–79^ and seems to mediate the level of consciousness^80, 81^. In short, stronger anticorrelations are thought to reflect a more effective capacity to switch between internal and external modes of attention^82^, with a self-related counterpart^83^. Therefore, the here-identified reductions in the anticorrelations may suggest a reduced ability for self-related monitoring during weightlessness.

It is possible that the observed changes in functional connectivity are due to a discrepancy between the gravitational vertical, as determined by integrated sensory information, and the expected vertical based on previous experience^84^. Such differences in experienced and expected spatial representations have also been estimated in off-vertical axis rotation^85^ and tilting train studies^86^. However, it remains challenging whether the observed effects can be attributed to microgravity, hypergravity or to the general transitions of the gravitational force, which are all induced during PF. With the current setup, we are unable to make specific assumptions as to the origin of the effect. Our control group did not engage in an activity that could mimic the characteristics of the PF, i.e. the alternation between the absence of gravity and the presence of hypergravity. Hence, we could not obtain a highly-controlled environment for the PF participants. Designs controlling for the effect of microgravity and the exposure to hypergravity, which is approximately twice the length of that spent in microgravity, might be able to disentangle between the effects of these two forces in the human brain. Finally, a potential confounder could be the mismatch in the time interval between scopolamine injection and post-scan sessions, which was on average 6 h for the PF group and 3 h for the non-PF group. We believe that this difference in scanning interval does not pose an issue on our analysis. This is because of the low dose of injected scopolamine in the non-PF group (0.175 mg for males, 0.125 mg for females), which was expected to washout after the 3 h interval. Based on a previous study assessing the effect of subcutaneous scopolamine (0.4 mg, 0.6 mg and 0.8 mg) on psychomotor tests, it was shown that scopolamine negatively affected the tests with a peak between 1–2 h after administration; after 3 h, values returned to baseline^87^. Here, the fact that we did not identify connectivity differences in the non-PF group between the post-pre scans, suggests a satisfactory baseline assessment for the PF group. Other fMRI studies, however, show that scopolamine affects functional connectivity^88–91^. In light of the different scanning setup, different dosage and administration type, it seems that results are not conclusive as to the exact affected regions. Also, even with scopolamine, motion sickness during PFs can still be present^23, 92^. Here, PF participants showed relatively low motion sickness scores. Additionally, they reported decreases in the negative affect as measured on subscale of the PANAS questionnaire after PF. This effect can be related to the fact that participants were generally excited by experiencing weightlessness. At the same time, we found a decrease in positive affect in the control group, which could be possibly related to boredom as reported in a previous study^93^. These confounds as well as the fact that a PF is associated with high stress levels and increases in stress hormones^94–96^ should be taken into consideration by future investigations.

In conclusion, we found that exposure to short-term acute alterations of gravity induced by a PF led to decreased intrinsic connectivity strength in the rAG/rTPJ, a region known to be involved in multisensory integration, cognitive and spatial tasks. Decreases in short-distance (within-hemisphere) and long-distance (inter-hemispheric) anticorrelations between the rAG/rTPJ and bilateral SMG were further identified. These results are relevant for long-duration spaceflight, as well as for space tourism, where less-trained humans will be exposed to similar and even more extreme gravitational transitions. Taken together, our findings shed light not only on the understanding of how the brain is affected by short-term alteration of gravitational input and the internalization of gravity in the human brain, but are also relevant for the understanding of bodily self-consciousness.

## Electronic supplementary material

Supplementary Online Methods
