# Supplementary material for: Intrinsic functional connectivity reduces after first-time exposure to short-term gravitational alterations induced by parabolic flight
Source: Sci Rep. 2017 Jun 12;7:3061. doi: 10.1038/s41598-017-03170-5 (PMC5468234; doi:10.1038/s41598-017-03170-5)

Supplementary Online material for

Intrinsic functional connectivity reduces after first-time exposure to short-term gravitational alterations induced by parabolic flight

Angelique Van Ombergen[1], Floris L. Wuyts[1], Ben Jeurissen[2], Jan Sijbers[2], Floris Vanhevel[3], Steven Jillings[1], Paul M. Parizel[3], Stefan Sunaert[4], Paul H. Van de Heyning[1], Vincent Dousset[5], Steven Laureys[6*], Athena Demertzi[6,7*]

* contributed equally

[1]Antwerp University Research Centre for Equilibrium and Aerospace (AUREA), University of Antwerp, Belgium

[2]Vision Lab, Department of Physics, University of Antwerp, Belgium

[3]Department of Radiology, Antwerp University Hospital & University of Antwerp, Belgium

[4]KU Leuven – University of Leuven, Department of Imaging & Pathology, Translational MRI, Leuven, Belgium

[5]University of Bordeaux, CHU de Bordeaux, INSERM Magendie, Bordeaux, France

[6]Coma Science Group, GIGA-Research & Neurology Department, University and University Hospital of Liège, Belgium

[7]Institut du Cerveau et de la Moelle Epinière - Brain and Spine Insititute, Hôpital Pitié-Salpêtrière, Paris, France

Corresponding author: Floris Wuyts, Antwerp University Research centre for Equilibrium and Aerospace (AUREA), University of Antwerp, Wilrijkstraat 10 (route 71), B-2650 Edegem (Antwerp), Belgium**.** Tel: +32 (0)3 821 47 10, E-mail : floris.wuyts@uantwerpen.be

Supplementary Online Methods

Due to the unbalanced design, two supplementary analyses were performed in order to increase statistical power and ensure validity and interpretation of the results: i) whole-brain connectivity analysis with the intrinsic connectivity contrast (ICC) between 12 PF and 12 non-PF subjects, matched for age (mean age 26y±3 SD and mean age 24y±3 SD respectively), and gender (4 females) and ii) bootstrapping.

Bootstrapping allows statistical inference by resampling sample data and performing population-level inference from these resampled observations. Here, we estimated random sampling distributions with replacement for the PF and non-PF groups and checked for statistical significance of the identified regions for the post-pre contrast: a) we subtracted the individual maps of whole-brain ICC connectivity at pre- from those maps at post-flight for each subject; b) with random selection, we chose 10 subjects from the PF and 10 subjects from the non-PF group; c) by 10,000 iterations, the mean ICC voxel-wise value was computed for each bootstrap sample; d) the probability that the mean distribution for the PF group was larger than the mean distribution of the non-PF group was estimated as counting the number of instances (i.e. iterations) in which the PF mean was larger than the non-PF mean; c) an empirical p-value for each voxel was constructed by dividing the above count by the total number of iterations (in those cases in which the total number was 0, it was set to the minimum theoretically possible p-value, i.e. 1/10000).

We found that the effect over the rAG was present both after the analysis described in the main manuscript and after controlling for gender and age. Bootstrapping further confirmed that the two groups had no occurrences of overlapping distributions over this area (Supplementary Fig. 1).

Supplementary Figure 1

The right angular gyrus/temporo-parietal junction is identified as the region with lower scores on the intrinsic connectivity contrast across multiple sub-analyses. The maps illustrate the clusters containing mean connectivity values after (A) including all subjects in the parabolic flight (PF) and the non-PF group and after (B) controlling for age and gender. Bars indicate effect sizes (beta values) and error bars 90%CI in the same cluster. (C) Bootstrapping further confirmed that the two groups had no occurrences of overlapping distributions over this area. The bootstrapping probability map (green cluster) has been corrected for multiple comparisons (FDR $p<0.05$).

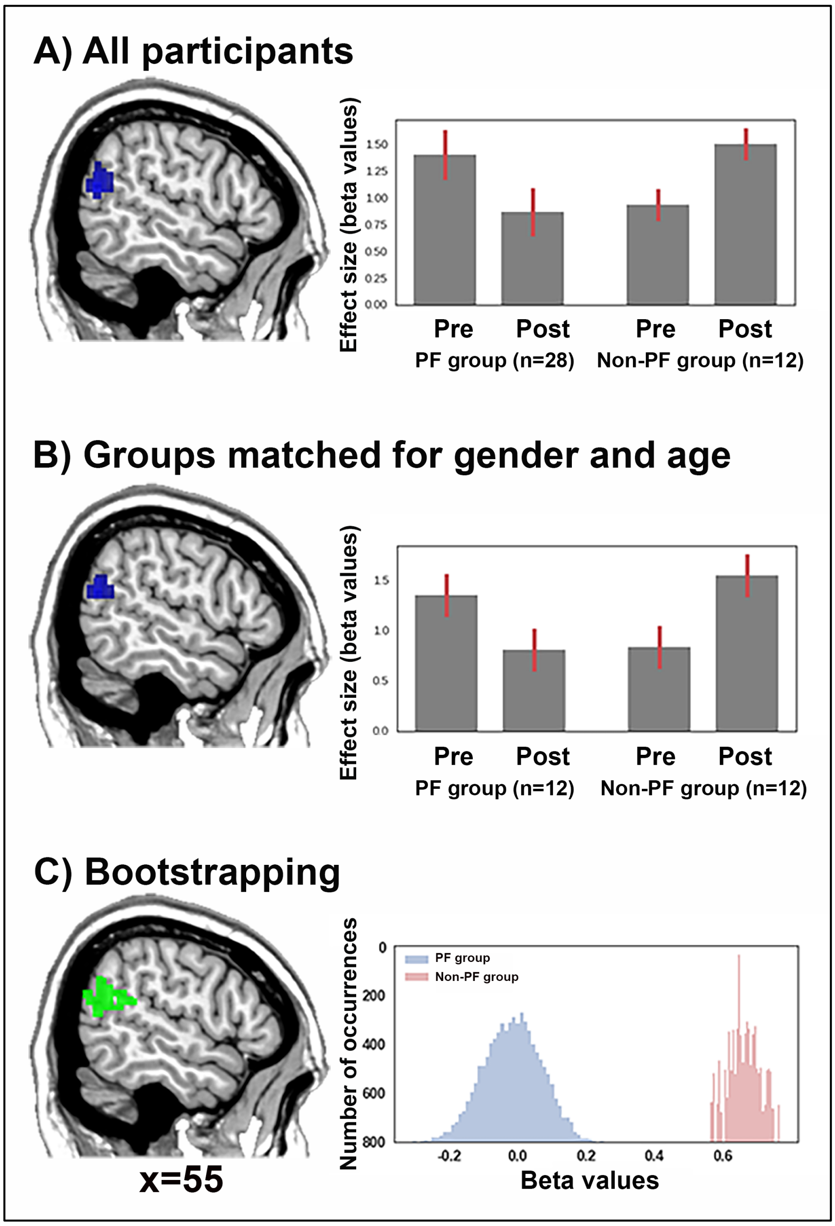

Supplement: Supplementary file 1 — Supplementary Online Methods [file 41598_2017_3170_MOESM1_ESM.pdf]
